# Identifying Reproducible Molecular Biomarkers for Gastric Cancer Metastasis with the Aid of Recurrence Information

**DOI:** 10.1038/srep24869

**Published:** 2016-04-25

**Authors:** Mengyao Li, Guini Hong, Jun Cheng, Jing Li, Hao Cai, Xiangyu Li, Qingzhou Guan, Mengsha Tong, Hongdong Li, Zheng Guo

**Affiliations:** 1 Key Laboratory of Ministry of Education for Gastrointestinal Cancer, Department of Bioinformatics, Fujian Medical University, Fuzhou 350001, China

## Abstract

To precisely diagnose metastasis state is important for tailoring treatments for gastric cancer patients. However, the routinely employed radiological and pathologic tests for tumour metastasis have considerable high false negative rates, which may retard the identification of reproducible metastasis-related molecular biomarkers for gastric cancer. In this research, using three datasets, we firstly shwed that differentially expressed genes (DEGs) between metastatic tissue samples and non-metastatic tissue samples could hardly be reproducibly detected with a proper statistical control when the metastatic and non-metastatic samples were defined by TNM stage alone. Then, assuming that undetectable micrometastases are the prime cause for recurrence of early stage patients with curative resection, we reclassified all the “non-metastatic” samples as metastatic samples whenever the patients experienced tumour recurrence during follow-up after tumour resection. In this way, we were able to find distinct and reproducible DEGs between the reclassified metastatic and non-metastatic tissue samples and concordantly significant DNA methylation alterations distinguishing metastatic tissues and non-metastatic tissues of gastric cancer. Our analyses suggested that the follow-up recurrence information for patients should be employed in the research of tumour metastasis in order to decrease the confounding effects of false non-metastatic samples with undetected micrometastases.

Tumour metastasis is the primary cause of recurrence and mortality of early stage gastric cancer patients after curative surgery^1^,^2^,^3^,^4^. Therefore, accurate diagnosis of distant and lymph node metastasis is essential for predicting prognosis and tailoring treatment strategies for gastric cancer patients^5^,^6^. However, current preoperative imaging techniques such as computed tomography (CT) and endoscopic ultrasound (EUS) are lack of accuracy^7^,^8^ and especially tend to produce a high rate of false negative clinical reports due to the poor identification of tiny lesions or micrometastases^4^,^7^,^8^. The lymph node metastasis is routinely detected by hematoxylin-eosin (H&E) staining of one section containing the largest dimension of the lymph node^6^, which also tends to produce a high rate of false negative clinical reports because of the random distribution of tumour cells throughout the lymph node^4^,^6^,^9^,^10^. More lymph node sections may decrease the false negative rate of H&E staining but the workload of surgeons and pathologists will be increased greatly^4^,^6^. The same problem exists when immunohistochemistry is used for detecting lymph node metastasis^4^,^6^. Consequently biomarkers for predicting the metastasis state for individual patients are in urgent need to avoid over- or inadequate-treatment owing to the misdiagnosis.

Because gene expression profiling has the advantage of exploring the tumour progression systematically based on the multiple gene disorders, many researches have exploited the high throughout data to study the transcriptional characteristics of metastasis and identify transcriptional biomarkers for metastasis^11^,^12^,^13^. Epigenomics data has also been taken into consideration by researchers and some methylation loci related to gastric metastasis have been reported^14^,^15^. However, the results of different studies showed inconsistency and lacked independent validation^16^. The same irreproducibility problem may exist for the basic task of extracting differentially expressed genes (DEGs) between the metastasis and non-metastasis samples^17^, which might make it unreliable to investigate the metastasis based on DEGs.

In this research, using three datasets of gene expression profiles for gastric cancer, we firstly showed that DEGs between metastatic tissue samples and non-metastatic tissue samples could hardly be reproducibly detected with a proper statistical control when the metastasis and non-metastasis samples were defined by TNM stage alone. Because micrometastases not found by the routine pathology diagnosis could be the major cause for recurrence after curative surgery^18^,^19^,^20^, we could hypothesize that the patients diagnosed as non-metastasis cases but subsequently suffered the recurrence should have developed micrometastases before the surgery. According to this hypothesis, we reclassified all the “non-metastatic” samples of patients, defined according to TNM stage, as metastatic samples whenever the patients experienced tumour recurrence during follow-up after tumour resection. By this strategy, we were able to find distinct and reproducible DEGs and concordant DNA methylation alterations between the reclassified metastatic and non-metastatic tissue samples with a proper statistical control false discovery rate (FDR) of less than 20%.

## Results

### Detecting reproducible metastasis-associated DEGs with the recurrence information

The TNM stage of the samples in the three datasets analysed in this study were diagnosed according to the 6th edition (GSE15459 and GSE62254) or 7th edition (TCGA batch 220) of the AJCC Cancer Staging Manual^21^, where the two editions have the same definition for metastasis and non-metastasis. According to TNM stage, the non-metastasis group consisted of samples without lymph node metastasis (N0) nor distant metastasis (M0), while the metastasis group included samples with lymph node metastasis (N+) and /or distant metastasis (M+) (Table 1). Using Wilcoxon rank-sum test with FDR < 10%, 126 and 1687 DEGs were detected between the metastasis group and non-metastasis group for the GSE15459 and GSE62254 datasets, respectively. The two lists of DEGs shared only 7 genes and the concordance score (see Materials and Methods) was 57.1% (*p* = 0.5). With FDR < 10%, 69 DEGs were found in TCGA batch 220 by the edgeR package (see Materials and Methods), of which only 1 and 3 DEGs were shared by GSE15459 and GSE62254, respectively (Supplementary Table 1). With FDR < 20%, 660 and 3371 DEGs were detected in GSE15459 and GSE62254 respectively. The two lists of DEGs had only 94 overlapped genes, and the concordance scores was as low as 54.3% (*p* = 0.24). With FDR < 20%, 124 DEGs were detected in the TCGA batch 220, of which only 3 and 15 DEGs were also detected as DEGs in GSE15459 and GSE62254 and the concordance scores were as low as 0% and 33.3% (*p* = 0.94), respectively. The low concordance scores and small overlaps between DEGs identified from independent datasets indicated that differential gene expression signals were weak and poorly reproducible in the three datasets when the samples were grouped by the TNM stage only (Supplementary Table 2), possibly due to confounding factors such as false negatives and/or false positive samples.

Considering that micrometastases undetectable by the routine pathology diagnosis could be the major cause for recurrence after curative surgery, we reclassified the samples by taking into account the recurrence information. GSE15459 provided the adjuvant treatment information for individual patients. Some patients experiencing no recurrence after curative surgery might benefit from the adjuvant treatment. Accordingly, only patients who were diagnosed as non-metastasis (N0M0) and did not recur under the condition of without adjuvant treatment were defined as the non-metastasis group. Because the information on adjuvant treatment were not explicitly provided in both GSE62254 and TCGA batch 220, the non-metastasis samples were defined as the ones who were diagnosed as non-metastasis (N0M0) and did not recur. For all these three datasets, the metastasis group consisted of the patients who were diagnosed as distant metastasis and the ones without distant metastasis but suffered from recurrence. In order to exclude the potential non-distant metastasis samples (false positive samples), we ignored the samples who were diagnosed as lymph node metastasis (N + M0) but did not recur after curative resection. After this reclassification, we obtained 94 metastasis samples and 27 non-metastasis samples in GSE15459, 132 metastasis samples and 27 non-metastasis samples in GSE62254, 18 metastasis samples and 11 non-metastasis samples in TCGA batch 220 respectively (Table 1). With FDR < 20%, the DEGs between the regrouped metastasis and non-metastasis samples were separately detected by the Wilcoxon rank-sum test for the GSE15459 and GSE62254 datasets and by the edgeR package for the data of the TCGA batch 220. After the sample reclassification, both the overlaps and concordance scores between every two lists of DEGs identified from the three independent datasets increased greatly (Supplementary Table 3). For GSE62254 and TCGA batch 220, the concordance score increased to 92.9% (*p* < 2.20 × 10^−16^) and the consistent DEGs increased to 2625. The concordance score between GSE15459 and GSE62254 increased to 90.5% (*p* < 1.11 × 10^−4^) and the score between the GSE15459 and TCGA batch 220 increased to 92.3% (*p* < 1.71 × 10^−3^), respectively, although they still had small numbers of overlapped DEGs. The high and statistically significant concordance scores verified that the reclassification of recurred samples was a powerful practice to extract reliable DEGs related to gastric cancer metastasis (Table 2). Part of the misjudged samples had been regrouped in accord with their actual metastasis status by this practice.

Functional enrichment analysis further supported that the DEGs consistently detected in GSE62254 and TCGA batch 220 were correlated with metastasis. With FDR < 20%, the DEGs up-regulated in the metastasis samples compared with the non-metastasis samples were significantly enriched in some typical tumour metastasis-associated signalling pathways, such as ECM-receptor interaction^22^, focal adhesion^23^,^24^,^25^ and cGMP-PKG signalling pathways^26^,^27^ (Supplementary Table 4). In contrast, the DEGs down-regulated in the metastasis samples were significantly enriched in pathways involved in cell metabolism, such as biosynthesis of amino acids, carbon metabolism, pyrimidine metabolism and many other pathways, such as homologous recombination, DNA replication and mismatch repair (Supplementary Table 4).

### Distinct epigenomic characteristics of metastasis

After reclassifying the samples with methylation data of TCGA batch 220 by the same rule used for the gene expression profiles, we compared the methylation profiles between the metastasis and the non-metastasis samples. Using the Wilcoxon rank-sum test with FDR < 20%, 447 and 233 genes were found to be hypermethylated and hypomethylated in the metastasis samples compared with the non-metastasis samples, respectively. Among the 447 hypermethylated genes, 62 genes were also identified as DEGs between the two groups, among which 90.3% were concordantly down-regulated in the metastasis samples compared with the non-metastasis samples, which was unlikely to be observed by chance (*p* < 1.49 × 10^−11^). These results suggested that hypermethylation of gene promoters may play a major role in inducing gene down-regulations in the metastasis tissues, and thus could be a major driver for the gastric cancer metastasis. Some of the concordant genes play important roles in the process of tumour cell migration. For example, IFNG in the regulation of autophagy pathway, which was both hypermethylated and down-regulated in metastasis tissues, might reduce cell epithelial apoptosis and decrease cell proliferation via autophagy^28^.

Similarly, 207 out of 233 hypomethylated genes were identified as DEGs between the two groups, among which 57.5% were concordantly up-regulated in the metastasis samples compared with the non-metastasis samples, which was also unlikely to be observed by chance (*p* < 0.02). Although the correlation between hypomethylation of gene promoters and gene overexpression was weak, DNA hypomethylation might also play a role in the metastasis. For example, we found that the COL4A3 annotated in the ECM-receptor interaction pathway was both hypomethylated and up-regulated in metastasis tissues, which might play a role in tumour metastasis^29^.

## Discussion

Our analyses demonstrated that the metastasis-associated differential gene expression signals were very weak and thus poorly reproducible in independent data when the samples were classified simply according to the TNM stage. The high recurrence rates of non-metastasis samples used in this study indicated high false negative rates, which might blur the difference between the metastasis and non-metastasis samples. This problem might exist for many studies on cancer metastasis mechanisms or predictive signatures, including both the high- and low-throughput researches. In order to reduce the interference of the false negative samples, we suggest making use of follow-up information of samples when researches on gastric cancer metastasis are conducted. Our results showed that distinct metastasis-associated DEGs could be reproducibly detected in independent data when the samples were regrouped based on both the TNM stage and recurrence information. With the help of recurrence information, classical metastasis-associated pathways significantly enriched with metastasis-associated DEGs could be readily detected, including focal adhesion, ECM-receptor interaction and metabolism pathways. The functional analysis results also provided extra evidence for the authenticity of the metastasis-associated DEGs identified between the reclassified metastasis and non-metastasis groups.

Gene expression alterations are usually caused by epigenomic and/or genomic lesions^30^,^31^. Metastasis-associated DNA methylation alterations which were significantly concordant with differential gene expressions were indeed observed between the reclassified metastasis and non-metastasis groups. Our results showed that hypermethylation of CpG loci in genes’ promoter regions could contribute to genes’ down-regulations in metastasis samples, indicating that DNA methylation alternation might be an important factor promoting cancer metastasis. However, we were unable to detect copy number alternations and gene mutations with significantly different frequencies between the metastasis and non-metastasis samples by the Fisher’s exact test with FDR control (FDR < 20%). The failure in finding genomic events characterizing the metastasis samples might indicate the existence of a certain percentage of misjudged samples in the datasets analysed in this study even after reclassifying some potential false negative samples. The undetected misjudged samples could be possibly due to the confounded effect of adjuvant treatment and short-term follow-up.

In summary, the false negative problem lays a major barrier for detecting reproducible metastasis-associated DEGs, let alone the identification of signatures for predicting metastasis. The same problem should exist in studies for other cancers and thus we suggest that the follow-up information should be taken into consideration for studying cancer metastasis.

## Materials and Methods

### Data acquisition and pre-processing

Gastric cancer gene expression profiles of the GSE15459 and GSE62254 datasets were downloaded from the GEO. The raw data (.CEL files) were normalized using the robust multi-array average method (RMA) in the Bioconductor package^32^,^33^,^34^. If multiple probes were mapped to the same gene, the expression value for the gene was summarized as the arithmetic mean of the values of the multiple probes (on the log2 scale). After data preprocess, 20283 genes were remained for analysis for both GSE15459 and GSE62254.

The multi-omic data for gastric cancer were derived from The Cancer Genome Atlas (TCGA) (http://cancergenome.nih.gov/). In order to avoid the batch effect, we restricted our analysis to samples of batch 220 which had comprehensive clinical information. The count data of RNA-seq were downloaded from the TCGA Web Portal. After excluding the unknown transcripts, we kept the data of 22509 genes for the following analysis. The methylation beta-values of samples measured by the Infinium HumanMethylation450 platform were downloaded from the TCGA Web Portal. Because the correlation between gene body methylation and gene expression is not clearly understood until now^35^,^36^, we focused on analysing the 27,578 CpG loci within the promoters for 14,495 protein-coding genes, which were defined in the Infinium HumanMethylation27 platform^37^. It has been widely recognized that there is a negative correlation between the promoter methylation and transcription activity, especially the hypermethylation of CpG loci in a gene promoter could lead to silence in gene transcription^38^,^39^. After excluding the loci with missing values, 22,432 CpG loci within the promoters for 14,495 protein-coding genes were analysed. CNVs data of level 4 of the TCGA samples analysed by GISTIC 2.0 were downloaded from Firehose (https://confluence.broadinstitute.org/display/GDAC/Download). A total of 36 significant amplification peaks and 53 deletion peaks were obtained.

### Identification of DEGs and differentially methylated genes

The two-tailed Wilcoxon rank-sum test was used to select DEGs and differentially methylated (DM) genes between metastasis and non-metastasis samples for microarray data and methylation data^40^. The R package of edgeR^41^ for RNA-seq data was conducted to exact DEGs between two kinds of samples. All the *p* values in this paper were adjusted by the Benjamini-Hochberg FDR procedure^42^.

### Analysis of epigenetic data

Only the CpG loci within the gene promoters for 14,495 protein-coding genes, as defined in the Infinium HumanMethylation27 platform^37^, were analysed. If a gene had both hypermethylated and hypomethylated CpG loci, this gene was excluded from subsequent analysis^43^. A gene with at least one DM locus in its promoter was termed a DM gene. By comparing the mean beta values of DM CpG loci between metastasis and non-metastasis samples, we classified the DM genes as hypermethylated genes or hypomethylated genes.

### Concordance scores

Suppose a couple of DEGs lists extracted separately from two datasets shared *k* genes, of which *s* genes showed the same deregulation directions (up- or down-regulation). In this case the concordance score was calculated as *s/k* × 100%. This score was used to evaluate the consistence of DEGs extracted from independent datasets.

If *k* genes are both significantly altered down-regulated (or up-regulation) in gene expression and methylated in the metastasis samples, of which *s* genes were hypermethylated (or hypomethylated) and correspondingly down-regulated (or up-regulation), then the concordance score was calculated as *s/k* × 100%. This score was used to evaluate the concordance of hypermethylation (or hypomethylation) with down-regulation (or up-regulation).

The probability of observing a concordance score of *s/k* by chance was evaluated by the cumulative binomial distribution model as follows:


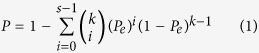


where *Pe* is the probability of one gene having the concordant relationship between the two lists of genes by chance (here, *Pe* = 0.5).

### Functional enrichment analysis

The functional enrichment analysis was conducted based on the Kyoto Encyclopedia of Genes and Genomes (KEGG) database^44^. The biological pathways in this database are described in KEGG Markup Language (KGML) files including nodes (genes and compounds) and edges (functional links). The KGML data files were obtained manually from the KEGG website in July, 2014. After removing the pathways without functional links between genes, we obtained 217 pathways. Functional KEGG enrichment analyses were performed separately for up- and down-regulated genes for the reason that it was more powerful than analysing all the DEGs together^45^. The biological pathways that were significantly enriched with genes of interest were determined by the hypergeometric distribution model. If *k* genes were identified as interesting genes (such as DEGs) from *n* genes in a dataset and *x* of them were annotated in a pathway with *m* genes, then the probability of observing at least *x* genes in this pathway by chance can be appropriately modelled by the cumulative hypergeometric distribution model as follows:


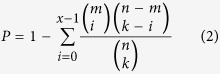


## Additional Information

**How to cite this article**: Li, M. *et al.* Identifying Reproducible Molecular Biomarkers for Gastric Cancer Metastasis with the Aid of Recurrence Information. *Sci. Rep.* **6**, 24869; doi: 10.1038/srep24869 (2016).

## Supplementary Material

Supplementary Information

## Figures and Tables

**Table 1:** Samples classified as metastasis and non-metastasis groups according to different criteria.

Dataset	Metastasis group	Non-metastasis group
Gene expression profiles (grouped by TNM stage)		
GSE15459	139	35
GSE62254	263	37
TCGA batch 220	33	19
Gene expression profiles (regrouped by TNM stage and recurrence information)		
GSE15459	94	27
GSE62254	132	27
TCGA batch 220	18	11
Methylation profile (regrouped by TNM stage and recurrence information)		
TCGA batch 220	21	14

**Table 2:** Concordance scores between DEGs detected from different datasets (FDR < 20%).

Datasets	TNM stage^1^		TNM stage and recurrence^2^	
	overlap(CS^3^)	*p* value	overlap(CS)	*p*value
GSE15459 vs. GSE62254	94 (54.3%)	0.24	21 (90.5%)	1.11 × 10^−4^
GSE62254 vs. TCGA batch 220	15 (33.3%)	0.94	2827 (92.9%)	<2.2 × 10^−16^
GSE15459 vs. TCGA batch 220	3 (0%)	>0.99	13 (92.3%)	1.71 × 10^−3^

Note: ^1^results for sample classified by the TNM stage alone.

^2^Results for sample classified by TNM stage and recurrence information.

^3^CS denotes for concordant score.
