# Supplementary material for: Identifying Reproducible Molecular Biomarkers for Gastric Cancer Metastasis with the Aid of Recurrence Information
Source: Sci Rep. 2016 Apr 25;6:24869. doi: 10.1038/srep24869 (PMC4843012; doi:10.1038/srep24869)
Supplement: Supplementary Information [file srep24869-s1.pdf]

Identifying Reproducible Molecular Biomarkers for Gastric Cancer Metastasis with the Aid of Recurrence Information

Mengyao Li, Guini Hong, Jun Cheng, Jing Li, Hao Cai, Xiangyu Li, Qingzhou Guan, Mengsha Tong, Hongdong Li, Zheng Guo

Contents

Supplementary Table 1

Supplementary Table 2

Supplementary Table 3

Supplementary Table 4

Supplementary Table 1. The concordance scores of DEGs from different datasets with samples grouped by TNM stage alone.

Datasets	DEGs 1*	DEGs 2	Overlap(CS)	*p* value
GSE62254 vs. TCGA batch 220	1687	69	3(33.3%)	0.88
GSE15459 vs. GSE62254	126	1687	7(57.1%)	0.5
GSE15459 vs. TCGA batch 220	126	69	1(0%)	>0.99

* DEGs were identified with FDR<10% for all datasets.

Supplementary Table 2. The concordance scores of DEGs from different datasets with samples grouped by TNM stage alone.

Datasets	DEGs 1*	DEGs 2	Overlap(CS)	*p* value
GSE62254 vs. TCGA batch 220	3371	124	15(33.3%)	0.94
GSE15459 vs. GSE62254	660	3371	94(54.3%)	0.24
GSE15459 vs. TCGA batch 220	660	124	3(0%)	>0.99

* DEGs were identified with FDR<20% for all datasets.

Supplementary Table 3. The DEGs and concordance scores of samples regrouped by TNM stage and recurrence information .

Datasets	DEGs 1*	DEGs 2	Overlap(CS)	*p* value
GSE62254 vs. TCGA batch 220	6731	7074	2827(92.9%)	$<2.20\times10^{-16}$
GSE15459 vs. GSE62254	68	6731	21(90.5%)	1.11×10^{-4}
GSE15459 vs. TCGA batch 220	68	7074	13(92.3%)	1.71×10^{-3}

* DEGs were identified with FDR<20% for all datasets.

Supplementary Table 4. Significant pathways enriched with DEGs identified consistently in GSE62254 and TCGA batch 220 (FDR<0.2).

KEGG_Pathway_ID	KEGG_Pathway_Name	N	n	m	k	Adjusted *p*
Pathways enriched from up-regulated genes						
hsa04512	ECM-receptor interaction	5573	344	87	21	3.85×10^{-8}
hsa04270	Vascular smooth muscle contraction	5573	344	116	24	9.93×10^{-8}
hsa04510	Focal adhesion	5573	344	206	32	8.37×10^{-7}
hsa04022	cGMP-PKG signalling pathway	5573	344	163	27	1.77×10^{-6}
hsa04360	Axon guidance	5573	344	126	22	6.90×10^{-6}
hsa04971	Gastric acid secretion	5573	344	74	14	1.37×10^{-4}
hsa04921	Oxytocin signalling pathway	5573	344	154	21	4.48×10^{-4}
hsa04261	Adrenergic signalling in cardiomyocytes	5573	344	146	19	1.47×10^{-3}

hsa04911	Insulin secretion	5573	344	83	13	1.55×10^{-3}
hsa04713	Circadian entrainment	5573	344	95	14	1.88×10^{-3}
hsa00982	Drug metabolism - cytochrome P450	5573	344	49	9	2.66×10^{-3}
hsa04020	Calcium signalling pathway	5573	344	178	21	2.92×10^{-3}
hsa04730	Long-term depression	5573	344	56	9	6.72×10^{-3}
hsa04710	Circadian rhythm	5573	344	29	6	7.49×10^{-3}
Pathways enriched from down-regulated genes						
hsa04110	Cell cycle	5573	570	123	46	4.44×10^{-16}
hsa03030	DNA replication	5573	570	36	21	1.45×10^{-12}
hsa03040	Spliceosome	5573	570	124	40	9.87×10^{-12}
hsa00970	Aminoacyl-tRNA biosynthesis	5573	570	44	19	1.39×10^{-8}
hsa03430	Mismatch repair	5573	570	22	12	2.89×10^{-7}
hsa03008	Ribosome biogenesis in eukaryotes	5573	570	74	22	2.40×10^{-6}
hsa03460	Fanconi anemia pathway	5573	570	49	17	3.29×10^{-6}
hsa03013	RNA transport	5573	570	148	32	2.68×10^{-5}
hsa03010	Ribosome	5573	570	122	28	2.72×10^{-5}
hsa00240	Pyrimidine metabolism	5573	570	97	24	2.76×10^{-5}
hsa01200	Carbon metabolism	5573	570	104	25	3.20×10^{-5}
hsa03050	Proteasome	5573	570	43	14	5.50×10^{-5}
hsa04115	p53 signalling pathway	5573	570	68	18	1.09×10^{-4}
hsa03420	Nucleotide excision repair	5573	570	42	13	1.81×10^{-4}
hsa03018	RNA degradation	5573	570	73	18	2.89×10^{-4}
hsa00670	One carbon pool by folate	5573	570	19	8	3.10×10^{-4}
hsa04114	Oocyte meiosis	5573	570	107	23	3.94×10^{-4}
hsa03410	Base excision repair	5573	570	33	10	1.21×10^{-3}
hsa04623	Cytosolic DNA-sensing pathway	5573	570	60	14	2.36×10^{-3}
hsa04621	NOD-like receptor signalling pathway	5573	570	57	13	4.13×10^{-3}
hsa04914	Progesterone-mediated oocyte maturation	5573	570	84	17	4.28×10^{-3}
hsa00190	Oxidative phosphorylation	5573	570	113	21	4.65×10^{-3}
hsa01230	Biosynthesis of amino acids	5573	570	71	15	4.66×10^{-3}
hsa03440	Homologous recombination	5573	570	28	8	5.55×10^{-3}
hsa00620	Pyruvate metabolism	5573	570	42	10	8.29×10^{-3}
hsa00020	Citrate cycle (TCA cycle)	5573	570	30	8	8.70×10^{-3}
hsa04650	Natural killer cell mediated cytotoxicity	5573	570	113	20	9.82×10^{-3}
hsa00010	Glycolysis / Gluconeogenesis	5573	570	65	13	1.30×10^{-2}
hsa00061	Fatty acid biosynthesis	5573	570	6	3	1.68×10^{-2}
hsa04612	Antigen processing and presentation	5573	570	56	11	2.43×10^{-2}
hsa00630	Glyoxylate and dicarboxylate metabolism	5573	570	23	6	2.47×10^{-2}

Note:

N: number of background genes; n: number of interesting genes; m: number of background genes in a pathway; k: number of interesting in a pathway; *p*: adjusted hypergeometric *p*-value.
